# P-1818. Pilot Testing of a Stakeholder-driven, Patient Education Intervention in Primary Care to Reduce Use of Antibiotics without a Prescription

**DOI:** 10.1093/ofid/ofae631.1981

**Published:** 2025-01-29

**Authors:** Tyler Brehm, Larissa Grigoryan, Kiara Olmeda, Azalia Mancera, Roger Zoorob, Mohamad Sidani, Lindsey Laytner, Mace Adams, Barbara Trautner

**Affiliations:** Baylor College of Medicine, Rosharon, TX; Baylor College of Medicine, Rosharon, TX; Baylor College of Medicine, Rosharon, TX; Center for Innovations in Quality, Effectiveness and Safety (IQuESt), Michael E. DeBakey Veterans Affairs Medical Center, Houston, Texas; Baylor College of Medicine, Rosharon, TX; Department of Family & Community Medicine, Baylor College of Medicine, Houston, Texas; Baylor College of Medicine, Department of Family and Community Medicine, Houston, TX; UT Health, Houston, Texas; Michael E. DeBakey Veterans Affairs Medical Center / Baylor College of Medicine, Houston, Texas

## Abstract

**Background:**

Antibiotic use without a prescription (non-prescription use) is a highly prevalent issue that poses significant individual and population level risk. We designed a patient-focused educational brochure to reduce non-prescription antibiotic use and report patient survey results about the brochure’s acceptability, appropriateness, and usability.

**Figure 1.**
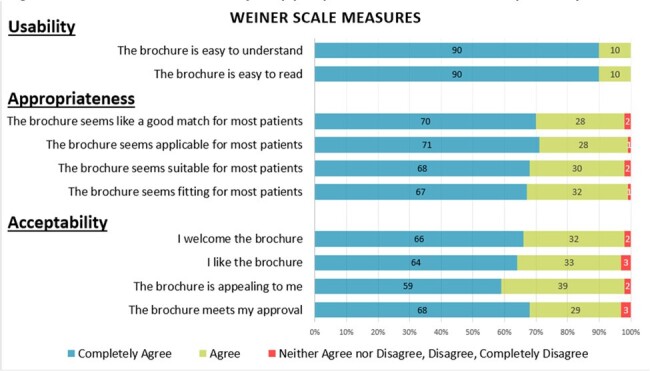
Brochure Usability, Appropriateness, and Acceptability

For the three criteria of usability, appropriateness, and acceptability, responses of "completely agree" and "agree" were categorized as positive while "neither agree or disagree," "disagree," or "completely disagree" were categorized as negative responses. n = 100 respondents.

**Methods:**

The brochure was developed with input from six racially/ethnically diverse community advisory board members plus 2 experts in health literacy and cultural competency in Hispanic communities. Brochure content included information on safe means of obtaining antibiotics, antibiotic side effects, non-antibiotic treatment of common symptoms, and how to access care at the public clinics. From October 2023 to February 2024, brochures (in English or Spanish) were distributed to 100 patients at two local clinics (50 each). Patients completed a survey about the brochure acceptability, appropriateness, and usability, followed by an open-ended question about suggestions for improvement.

**Results:**

Survey participants were on average 53 years old, 72% female, 72% college educated, and primarily Black (35%), Hispanic or Latino (30%), or Caucasian (28%). 81% had adequate health literacy. Respondents largely found the brochure acceptable (98%), appropriate (98%), and usable (100%). 96% of respondents said the brochure was useful, referencing learning about antibiotic indications (23%), over the counter alternatives for symptom relief (23%), and antibiotic side effects (14%). The most common reason respondents did not find the brochure useful was they already “knew everything in [the] brochure” (n = 4). 15 respondents (15%) had suggestions for improvement such as having an alternative media form (e.g., an audio version) and having the brochure available at the pharmacy (27% each).

**Conclusion:**

We designed a brochure about antibiotic safety that was considered acceptable, appropriate, and usable by our diverse clinic patients. Key learning points reported by patients included antibiotic indications, side effects, and over the counter alternatives. Next the brochure will be the focus of an intervention to reduce non-prescription antibiotic use among our socially vulnerable patient population.

**Disclosures:**

**Barbara Trautner, MD, PhD**, Abbott Laboratories: Stocks/Bonds (Public Company)|AbbVie: Stocks/Bonds (Public Company)|Bristol Myers Squibb: Stocks/Bonds (Public Company)|Pfizer: Stocks/Bonds (Public Company)|Phiogen Pharma: Advisor/Consultant

